# Effect of programmed cell death protein-1 inhibitor combined with platinum-containing dual-agent chemotherapy regimen on gut microbiota in Lewis lung cancer model mice

**DOI:** 10.3389/fmicb.2026.1885048

**Published:** 2026-07-10

**Authors:** Li Dai, Fan-Lei Kong

**Affiliations:** 1 Department of Stomatology, Shandong Provincial Hospital Affiliated to Shandong First Medical University, Jinan, China; 2 Department of Radiology, Qilu Hospital of Shandong University, Jinan, China

**Keywords:** 16S rRNA gene, chemotherapy, gut microbiota, Lewis mice, PD-1

## Abstract

**Purpose:**

To explore the effects of programmed cell death protein-1 (PD-1) combined with pemetrexed (PEM) and carboplatin (CARB) chemotherapy regimen on the gut microbiota in the Lewis lung cancer model mice compared to chemotherapy alone.

**Materials and methods:**

C57BL/6 J male mice aged 10–12 weeks were selected to establish the Lewis lung cancer model by planting tumors in the right forelimb, and were randomly divided into negative control group (NC group), chemotherapy group (PEM-CARB group), and chemotherapy combined with immunotherapy group (PEM-CARB-PD-1 group), with eight mice in each group. The total RNA of fecal bacteria was collected from the feces of mice in each group after two cycles of drug administration. 16S rRNA gene amplification and high-throughput sequencing were performed to analyze the Alpha diversity, Beta diversity, composition, and function in the gut microbiota.

**Results:**

The Alpha diversity was not statistically different between the PEM-CARB-PD-1 group and the PEM-CARB group (Shannon index: *p* = 0.645; Simpson index: *p* = 0.879). The Beta diversity between the PEM-CARB-PD-1 group and PEM-CARB group was statistically different [weighted Unifrac Principal Co-ordinate Analysis (PCoA), *p* = 0.001; unweighted Unifrac PCoA, *p* < 0.001]. However, the Beta diversity between the PEM-CARB-PD-1 group and the NC group did not reveal statistical differences (weighted Unifrac PCoA, *p* = 0.690; unweighted Unifrac PCoA, *p* = 0.135). Compared to the PEM-CARB group, the combination of the PD-1-inhibitor affects both the “response-favorable taxa” and “response-unfavorable taxa” for immunotherapy. Notably, the PEM-CARB-PD-1 group had an increased abundance of Gram-positive bacterial phenotypes relative to the PEM-CARB group (*p* = 0.038). Nearly no statistically significant differences in metabolic pathways were seen between the PEM-CARB-PD-1 group and the PEM-CARB group.

**Conclusion:**

Combination therapy affects both “response-favorable taxa “and “response-unfavorable taxa associated with immunotherapy, and the ultimate impact remains dependent on the ratio of the two types of flora. Predicted metabolic pathway analysis using PICRUSt2 suggested that the combination regimen may not further reduce predicted functional pathway abundance beyond that observed with chemotherapy alone. However, these predictions require validation through direct metagenomic or metabolomic approaches.

## 1 Introduction

According to the Global Cancer Statistics 2020, lung cancer has the second highest incidence of all cancers and is the leading cause of death from all cancers, of which more than 80% are non-small cell lung cancer (NSCLC) (National-Comprehensive-Cancer Network, 2006; World Health Organization, 2020). With the development of precision cancer treatment, “immunotherapy” represented by immune checkpoint inhibitors (ICIs), following “targeted therapy” has gradually one of the important treatments for NSCLC. The U.S. Food and Drug Administration approved pembrolizumab in combination with pemetrexed/paclitaxel and platinum drugs for the first-line treatment of EGFR/ALK-negative metastatic NSCLC.

Gut microbiota plays an important role in the human immune system (Clemente et al., 2012; Belkaid and Hand, 2014; Hooper et al., 2012; Ivanov and Honda, 2012). And, the influence of gut microbiota on the efficacy of chemotherapy through immunomodulation has been widely recognized (Viaud et al., 2013; Daillere et al., 2016; Cortez-Retamozo et al., 2012; Mukaida, 2014; Gui et al., 2015). Recent studies have reported that gut microbiota can influence the host’s response to immunotherapy. Patients with better clinical response to PD-1 exhibit higher diversity and unique gut microbiota composition, and those patients have significantly enhanced peripheral memory T cell and natural killer cells, making gut microbiota a potential predictive biomarker for immunotherapy (Routy et al., 2018; Matson et al., 2018; Gopalakrishnan et al., 2018; Sivan et al., 2015; Carbone et al., 2019; Jin et al., 2019; Zhang et al., 2021).

The treatment of tumors can likewise affect the gut microbiota. Chemotherapy can induce an imbalance in gut microbiota, resulting in a reduction in population and diversity of intestinal bacteria and a change in the relative abundance of specific taxa (Sougiannis et al., 2019; Xu and Zhang, 2015; Panebianco et al., 2018; Yang et al., 2013). An imbalance of gut microbiota not only promotes the occurrence and development of tumors but also affects the efficacy and safety of tumor treatment, ultimately affecting patients’ survival and quality of life (Yuan et al., 2018).

Current immunotherapy-related studies have mostly focused on exploring the effect of gut microbiota on the efficacy of ICIs, and the impact of chemotherapy combined with PD-1 regimens on gut microbiota is lacking. In this study, we employed 16S rRNA gene amplicon sequencing to characterize the gut microbiota composition and predict functional profiles of Lewis lung cancer model mice treated with carboplatin/pemetrexed (AP regimen) alone or in combination with anti-PD-1 antibody. Our aim was to provide a descriptive characterization of microbiota structural changes associated with combination immunochemotherapy, generating hypotheses for future mechanistic and translational studies investigating the microbiota-immunotherapy axis in lung cancer.

## 2 Materials and methods

### 2.1 Animal model and study design

This study was approved by the Ethics Committee of Qilu Hospital of Shandong University and followed the animal care guidelines (Approval No. KYLL-2022(ZM)-1121). C57Bl/6 J mice were purchased from Beijing Weitonglikang Technology Company and housed at the Animal Experiment Center of Shandong University of Traditional Chinese Medicine in a Specific Pathogen Free (SPF)-grade environment. Mouse Lewis lung cancer cell line purchased from Shanghai Cell Bank of Chinese Academy of Sciences. Anti-mouse PD-1 monoclonal antibody (200 μg per mouse per dose; intraperitoneal injection; twice weekly; [manufacturer: Bio X Cell, clone: RMP1-14, Cat#: BE0146, Lot#: LVF288-3]). The PD-1 antibody is a rat anti-mouse PD-1 (CD279) monoclonal antibody specifically designed for *in vivo* use in mouse models.

Ten to twelve weeks C57BL/6 J male mice were inoculated with Lewis lung cancer cells subcutaneously in the axilla of the right forelimb (Reagan-Shaw et al., 2008; Van Damme et al., 2021). Mice with right forelimb tumors of 50-100 mm^3^ were randomly divided into NC group (*n* = 8), PEM-CARB group (*n* = 8) and PEM-CARB-PD-1 group (*n* = 8). Groups of mice were injected intraperitoneally weekly using the corresponding drugs. The types and doses of drugs used in each group were as follows:

PEM-CARB group: CARB 60.8 mg/kg/each, PEM 112.5 mg/kg/each.

PEM-CARB-PD-1 group: CARB 60.8 mg/kg/each, PEM 112.5 mg/kg/each, PD-1200ug/each.

NC group: equal volume of saline.

Additionally, mice from the same batch but not subjected to modeling were used as the baseline group.

### 2.2 Fecal collection

Mice feces were collected in sterile freezing tube a day after the end of the second cycle of dosing and placed in a refrigerator at −80 °C until the analysis of microbiota composition was performed. Fecal samples of baseline group were collected before treatment initiation. To minimize contamination, collection was performed on clean, autoclaved bench surfaces, and gloves were changed between each mouse.

### 2.3 Microbial genomic DNA extraction

- Total microbial genomic DNA was extracted from fecal samples using the HiPure Stool DNA Kit (Magen, Guangzhou, China, Cat# D3141) according to the manufacturer’s instructions. DNA concentration was measured using a NanoDrop 2000 spectrophotometer (Thermo Fisher Scientific, USA), and DNA integrity was assessed by 1% agarose gel electrophoresis. Only samples with A260/A280 ratios between 1.8 and 2.0 and clear single bands on gel electrophoresis were used for subsequent analysis. All DNA extraction and PCR procedures were performed under sterile conditions with negative extraction controls.

### 2.4 16S rRNA gene amplicon sequencing

The V3–V4 hypervariable regions of the bacterial 16S rRNA gene were amplified using the following primers:

- Forward primer 341F: 5’-CCTACGGGNGGCWGCAG-3’
- Reverse primer 806R: 5’-GGACTACHVGGGTATCTAAT-3’

PCR amplification was performed in a 50 μL reaction system containing 5 μL of 10 × KOD buffer, 5 μL of 2.5 mM dNTPs, 1.5 μL of each primer (5 μM), 1 μL of KOD polymerase, and 100 ng of template DNA. Thermal cycling conditions were: initial denaturation at 95 °C for 2 min; 27 cycles of 98 °C for 10 s, 62 °C for 30 s, and 68 °C for 30 s; and a final extension at 68 °C for 5 min. PCR products were verified on 2% agarose gel and purified using AMPure XP Beads (Beckman Coulter, USA).

Purified amplicons were quantified using a QuantiFluor™ fluorometer (Promega, USA), pooled in equimolar amounts, ligated with sequencing adapters, and constructed into sequencing libraries. Libraries were sequenced on the Illumina NovaSeq 6,000 platform generating paired-end 250 bp reads (PE250). Sequencing was performed by Gene Denovo Biotechnology Co., Ltd. (Guangzhou, China).

Sequencing depth and quality control metrics for all samples are summarized in Supplementary Table S1.

### 2.5 Bioinformatics analysis

#### 2.5.1 Quality control and assembly

Raw paired-end reads were quality-filtered using FASTP (v0.18.0) to remove reads with average quality score < 20, reads containing ambiguous bases (N), and adapter sequences. Quality-filtered paired-end reads were assembled into tags using FLASH (v1.2.11) with a minimum overlap of 10 bp and a maximum mismatch rate of 2%. Assembled tags were further filtered to remove those with consecutive low-quality bases (quality score < 6 for ≥ 3 consecutive bases) to obtain Clean Tags.

#### 2.5.2 OTU clustering and taxonomic classification

Clean Tags were clustered into Operational Taxonomic Units (OTUs) at 97% sequence similarity using the UPARSE algorithm implemented in USEARCH (v9.2.64). Chimeric sequences were identified and removed using the UCHIME algorithm in USEARCH. The remaining Effective Tags were used for downstream analysis. Representative sequences from each OTU were taxonomically classified against the SILVA database (v138) using the RDP classifier with a confidence threshold of 0.8. Taxonomic assignments were made at the phylum, class, order, family, genus, and taxa levels.

#### 2.5.3 Alpha diversity analysis

Alpha diversity indices including Shannon, Simpson, Chao1, ACE, and observed taxa richness were calculated using QIIME2 (v2020.2). Rarefaction curves were generated to assess sequencing depth adequacy.

Beta diversity analysis: Beta diversity was assessed using Principal Coordinates Analysis (PCoA) based on four distance matrices: Weighted UniFrac, Unweighted UniFrac, Bray-Curtis, and Jaccard distances.

Functional prediction: Predicted functional profiles were inferred using PICRUSt2 (Phylogenetic Investigation of Communities by Reconstruction of Unobserved States, v2.3.0) based on 16S rRNA gene amplicon data and reference genomes from the Integrated Microbial Genomes (IMG) database. Functional pathways were mapped to KEGG orthology. Microbial community phenotypes were predicted using BugBase.

### 2.6 Statistical analysis

Statistical analysis was performed using SPSS 21.0 software (IBM SPSS software, Armonk, New York), or GraphPad Prism 6.0 (GraphPad software Inc., San Diego, California). The specific statistical methods applied were:

#### 2.6.1 Alpha diversity

Between-group comparisons of Shannon index, Simpson index, Chao1, ACE, and observed taxa were performed using the Wilcoxon rank-sum test (two-group comparisons) or Kruskal-Wallis test (multi-group comparisons). *p* < 0.05 was considered statistically significant.

#### 2.6.2 Beta diversity

Statistical significance of between-group differences was assessed using: (1) ANOSIM (Analysis of Similarity): A non-parametric test with 999 permutations. The R statistic indicates the degree of between-group vs. within-group difference (R > 0.75: well separated; 0.5–0.75: separated with overlap; 0.25–0.5: separated but overlapping; < 0.25: barely separable). *p* < 0.05 indicates significant difference. (2) Adonis (PERMANOVA, Permutational Multivariate Analysis of Variance): Performed with 999 permutations, reporting *R*^2^ (proportion of variance explained), *F*-statistic, and *p*-value. (3) PERMDISP (Permutational Analysis of Multivariate Dispersions): To test homogeneity of within-group dispersions and ensure that observed beta diversity differences reflect true structural changes rather than differences in group variability.

#### 2.6.3 Differential abundance analysis

LEfSe (Linear discriminant analysis Effect Size) was used with a multi-step approach: (1) Kruskal–Wallis test for initial multi-group screening, (2) Wilcoxon rank-sum test for pairwise comparisons, and (3) LDA score calculation (threshold: |LDA| > 2.0). For genus-level pairwise comparisons, Wilcoxon rank-sum tests were performed with Benjamini-Hochberg false discovery rate (FDR) correction for multiple testing. Adjusted *p* < 0.05 was considered significant.

#### 2.6.4 Functional predictions

Between-group differences in PICRUSt2-predicted KEGG pathways and BugBase phenotypes were assessed using Wilcoxon rank-sum tests with FDR correction.

## 3 Results

### 3.1 Diversity analysis of gut microbiota

#### 3.1.1 Alpha diversity

**Figure 1:**
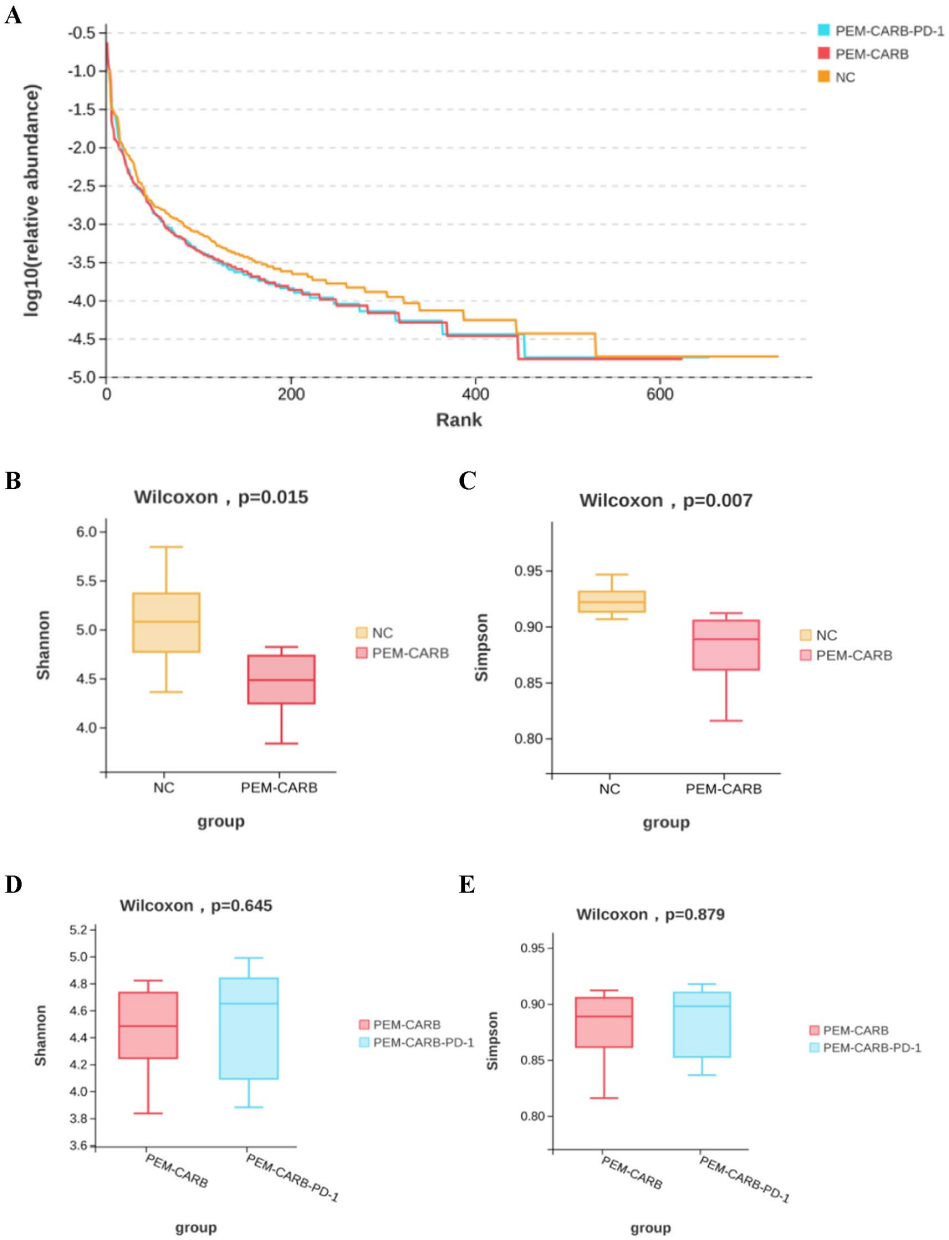
Comparison of Alpha diversity. **(A)** Rank abundance curves for Alpha diversity. The size of the span of the curve on the horizontal axis indicates the richness of the flora, and the degree of flattening of the curve indicates the homogeneity. The richness and homogeneity of the gut microbiota of Lewis mice in the NC group was higher than that in the PEM-CARB group and PEM-CARB-PD-1 group. **(B–E)** Box plot of statistical test for Alpha diversity. Compared with NC group, Shannon’s index nor Simpson’s index in PEM-CARB group were significantly decreased (Wilcoxon rank sum test, *p* = 0.015, *p* = 0.007, respectively). Neither Shannon’s index nor Simpson’s index was statistically different between the PEM-CARB-PD-1 group and the PEM-CARB group (Wilcoxon rank sum test, *p* = 0.645, *p* = 0.879, respectively).

In this study, Shannon and Simpson indices, which can reflect both the abundance and homogeneity of the flora, were chosen to assess the Alpha diversity of samples. As shown by the Rank abundance curves (Figure 1A), the abundance and homogeneity of gut microbiota of Lewis mice in the PEM-CARB group and PEM-CARB-PD-1 group were reduced compared to that of mice in the NC group. Both the Shannon and Simpson indices were significantly lower in the PEM-CARB group than in the NC group (PEM-CARB group vs. NC group: Shannon 4.445 ± 0.341 vs. 5.078 ± 0.465, *p* = 0.015; Simpson 0.880 ± 0.033 vs. 0.918 ± 0.026, *p* = 0.007) (Figures 1B,C),indicating that chemotherapy reduced microbial diversity. Notably, the Shannon and Simpson indices were not statistically different between the PEM-CARB-PD-1 group and the PEM-CARB group (PEM-CARB-PD-1 group vs. PEM-CARB group: Shannon 4.371 ± 0.689 vs. 4.445 ± 0.341, *p* = 0.645; Simpson 0.857 ± 0.097 vs. 0.880 ± 0.033, *p* = 0.879) (Figures 1D,E). Similar trends were observed for the Chao1 and ACE indices, suggesting that the addition of PD-1 inhibitor to chemotherapy did not further reduce taxa richness beyond the level observed with chemotherapy alone, although this observation requires cautious interpretation given the sample size.

The results of diversity of the gut microbiota in Lewis mice between baseline group and NC group was shown in Supplementary Figure 1.

#### 3.1.2 Beta diversity

**Figure 2:**
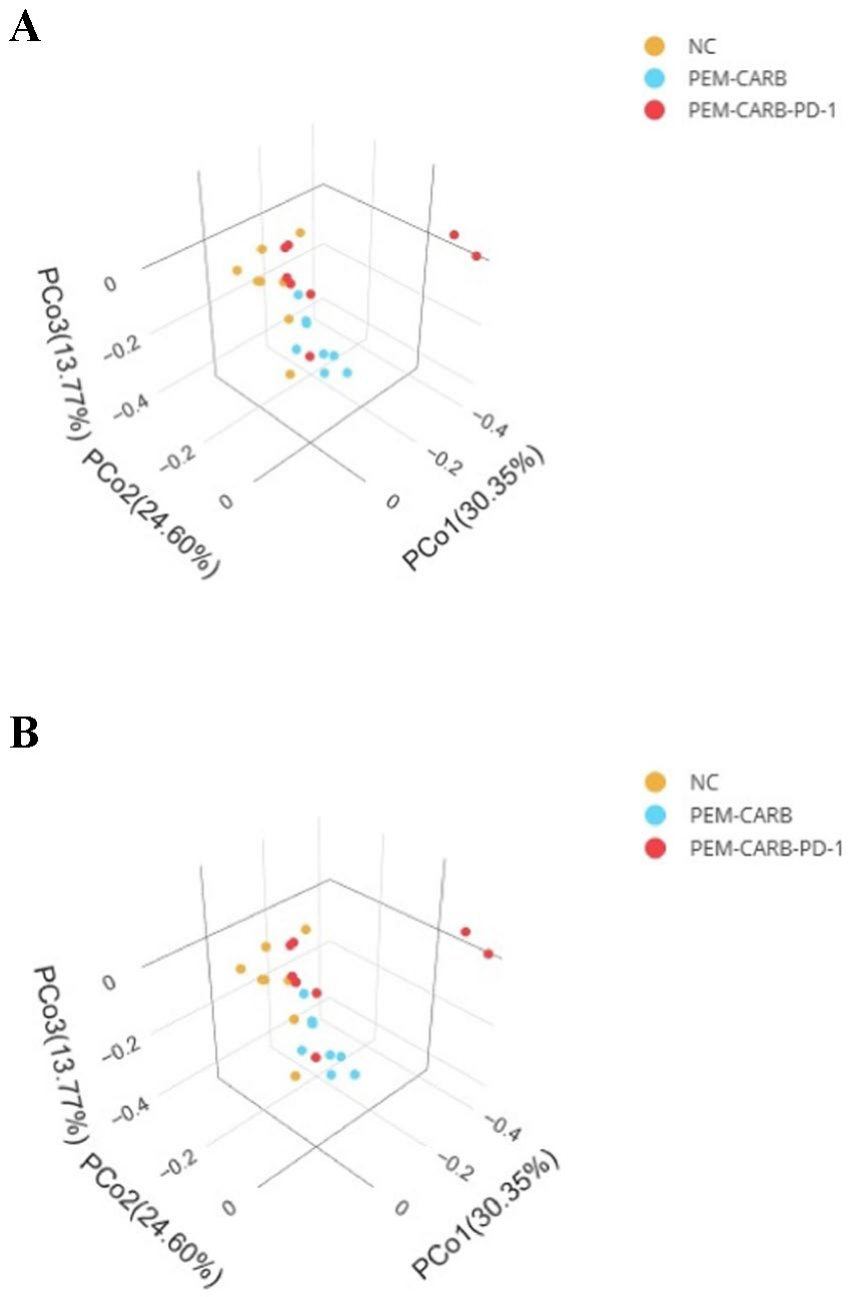
Comparison of Beta diversity. **(A)** Weighted Unifrac PCoA 3D scatterplot. **(B)** Unweighted Unifrac PCoA 3D. scatterplot. Each point in the graph represents a sample, and the closer the points are to each other on the plane, the more similar the colony structures of the samples are. PCoA assesses the degree to which each axis explains the overall differences in colony structure in terms of percentages (numbers in parentheses in the axes’ headings). As seen in the figure, the PEM-CARB-PD-1 group was structurally more similar to the NC group than to the PEM-CARB group, whether weighted or unweighted PCoA.

As for Beta diversity, this study used weighted and unweighted Unifrac PCoA to evaluate the similarity of the bacterial colony structure between different groups (Figures 2A,B). ANOSIM analysis confirmed statistically significant separation between the CARB-PEM group and the NC group (Unweighted UniFrac: *R* = 0.6295, *p* = 0.001; Weighted UniFrac: *R* = 0.5832, *p* = 0.001), and between the CARB-PEM group and the CARB-PEM-aPD-1 group (Unweighted UniFrac: *R* = 0.4096, *p* = 0.001). Adonis (PERMANOVA) analysis corroborated these findings: CARB-PEM vs. NC (*R*^2^ = 0.4521, *F* = 4.9452, *p* = 0.001); CARB-PEM-aPD-1 vs. CARB-PEM (*R*^2^ = 0.3365, *F* = 3.0412, *p* = 0.001). For the comparison between CARB-PEM-aPD-1 and NC groups, ANOSIM yielded *R* = 0.5926, *p* = 0.001 (Unweighted UniFrac), while Adonis showed *R*^2^ = 0.2808, *F* = 1.8374, *p* = 0.116. The discrepancy between ANOSIM and Adonis results suggests that while community rank-order differences exist, the proportion of variance explained by group membership is limited. These results indicate that both chemotherapy and combination therapy significantly altered gut microbial community structure relative to the untreated tumor-bearing state, and that the combination regimen produced a community structure distinguishable from that of chemotherapy alone.

### 3.2 Taxonomic composition

At the phylum level, the dominant phyla across all groups were Bacteroidetes, Firmicutes, Verrucomicrobia, Actinobacteria, and Proteobacteria. The CARB-PEM group showed a significantly increased relative abundance of the phylum Firmicutes and decreased relative abundance of the phylum Bacteroidetes compared to the Model group (FDR-adjusted *p* < 0.05). The CARB-PEM-aPD-1 group showed intermediate values.

**Figure 3:**
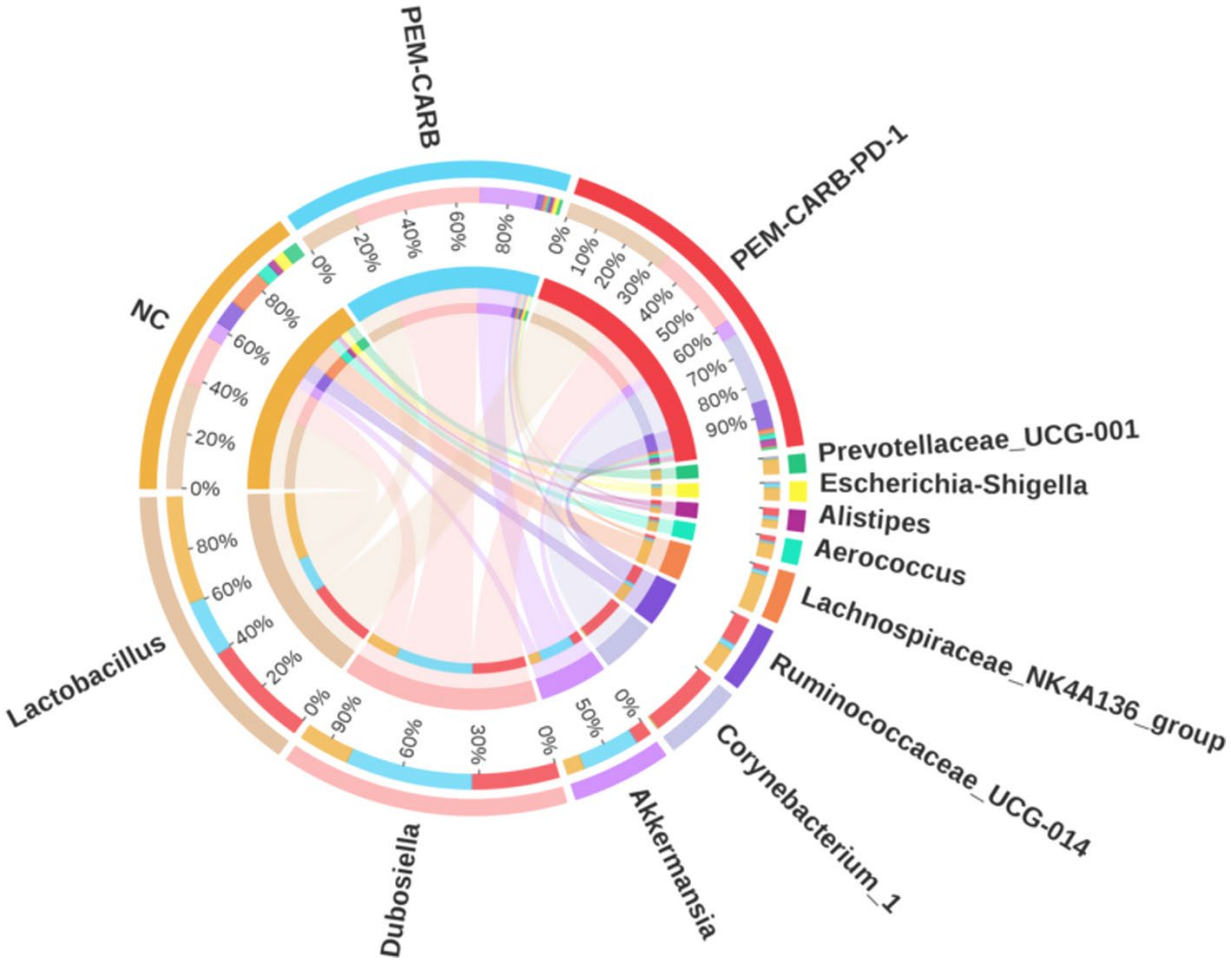
Circos diagram. One side of the graph is the group information, and the other side is the taxa information. The lines on both sides indicate a pair of corresponding relationships, and the thicker the lines, the larger the abundance value. The top 10 taxa were: Lactobacillus, Dubosiella, Akkermansia, Corynebacterium, Ruminococcacea, Lachnospiraceae, Aerococcus, Alistipes, Escherichia-Shigella, Prevotellaceae. Lactobacillus and Dubosiella had the highest average abundance in each group, while Akkermansia had relatively higher abundance in the PEM-CARB group, and Corynebacterium had the highest relative abundance in the PEM-CARB-PD-1 group.

At the genus level, the top 10 genera were: Lactobacillus, Dubosiella, Akkermansia, Corynebacterium, Ruminococcacea, Lachnospiraceae, Aerococcus, Alistipes, Escherichia-Shigella, Prevotellaceae (Figure 3).

Among the above genera, the abundance of Dubosiella (*p* = 0.002) was significantly higher in the PEM-CARB group than in the NC group, while the abundance of Lactobacillus (*p* = 0.049), Lachnospiraceae (*p* = 0.021), Prevotellaceae (*p* = 0.002), and Ruminococcus (*p* = 0.028) was significantly lower than in the NC group. Notably, the present study found a higher relative abundance of Akkermansia, which is thought to be beneficial for immunotherapy, in the PEM-CARB group than in the NC group, though the difference did not appear statistically significant (*p* = 0.130). Compared with the NC group, the abundance of Corynebacterium (*p* < 0.001) was significantly higher in the PEM-CARB-PD-1 group, whereas the abundance of Lachnospiraceae (*p* = 0.015), Ruminococcus (*p* = 0.007) and Prevotellaceae (p < 0.001) was significantly reduced. The abundance of Corynebacterium (*p* = 0.006), Lactobacillus (*p* = 0.028), and Ruminococcus (*p* = 0.006) was significantly higher in the PEM-CARB-PD-1 group than in the PEM-CARB group. In contrast, Prevotellaceae abundance was significantly reduced in the PEM-CARB-PD-1 group relative to the PEM-CARB group (*p* = 0.010).

We note that these taxonomic differences are described at genus or family level and do not permit inference about taxa- or strain-level functional properties.

The result of species distribution stacking maps at the genus level between baseline group and NC group was shown in Supplementary Figure 2.

### 3.3 Functional analysis

#### 3.3.1 Phenotype prediction

**Figure 4:**
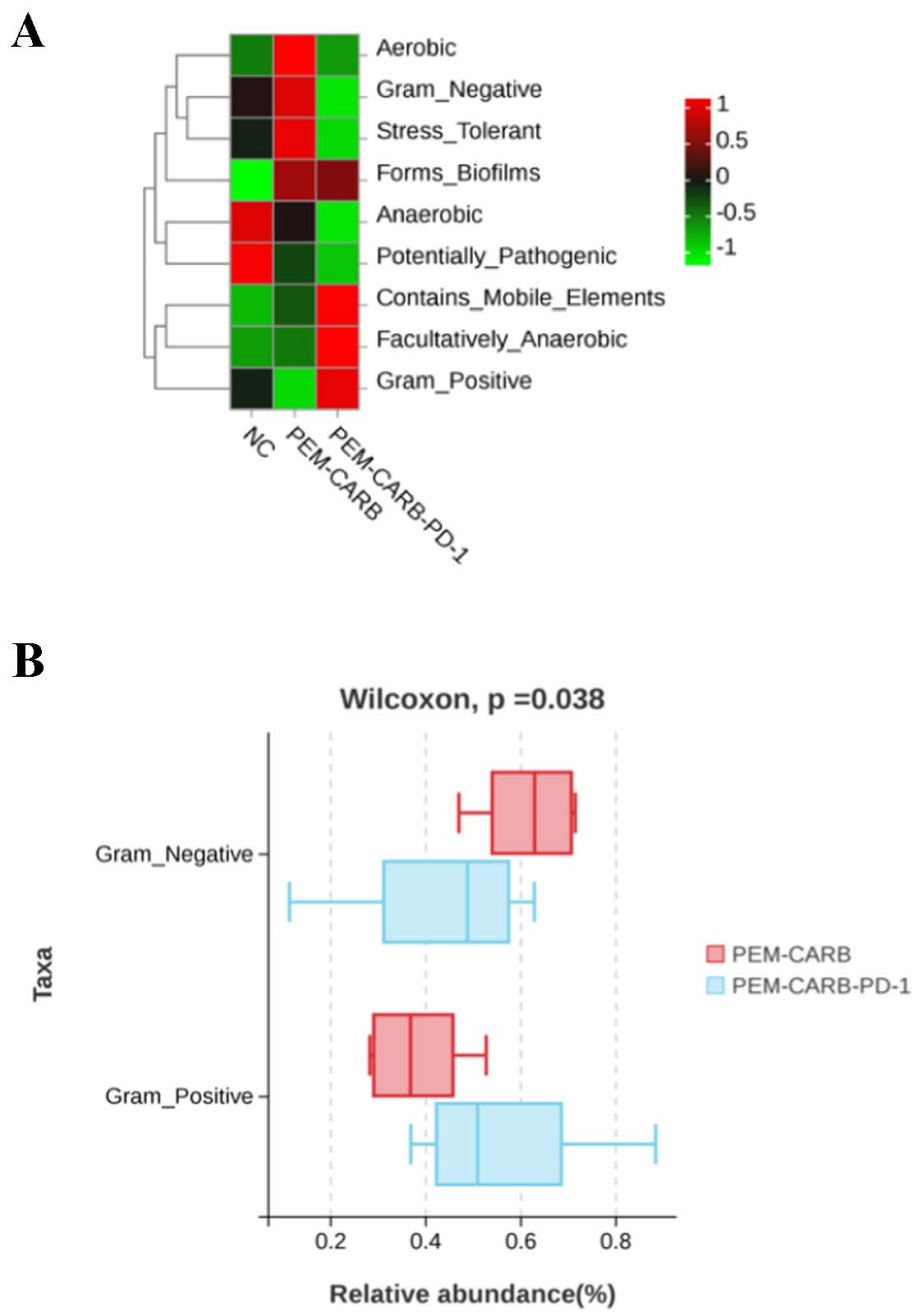
Comparison of function. **(A)** Heatmap of phenotypic abundance. The phenotypic information of taxa was classified into Gram Positive, Gram Negative, Biofilm Forming, Pathogenic, Mobile Element Containing, Aerobic, Anaerobic, Facultatively anaerobic, and Oxidative Stress Tolerant. **(B)** Box plot of statistical test for phenotypic abundance. The PEM-CARB-PD-1 group showed increased phenotypic abundance of Gram-Positive bacteria and decreased Gram-negative bacteria phenotypic abundance compared to the PEM-CARB group (Wilcoxon rank sum test, *p* = 0.038).

In terms of phenotype prediction, based on the genetic information of taxa in three major databases, Integrated Microbial Genomes (IMG), Kyoto Encyclopedia of Genes and Genomes (KEGG), and The Pathosystems Resource Integration Center (PATRIC), this research classified the phenotypic information of taxa into Gram positive, Gram negative, biofilm forming, pathogenic, mobile element containing, aerobic, anaerobic, facultatively anaerobic, oxidative stress tolerant, and predicted differences in the phenotypic abundance of Lewis mice gut microbiota after treatment in each group using BugBase software (Figure 4A).

BugBase-predicted phenotype analysis revealed no significant difference in the prediction of bacterial phenotype between the PEM-CARB group and the NC group. Notably, the PEM-CARB-PD-1 group had an increased abundance of Gram-positive bacterial phenotypes relative to the PEM-CARB group (*p* = 0.038), whereas the Gram-negative bacterial phenotypes had a decreased abundance (*p* = 0.038), and the abundance phenotypes of oxidative stress tolerant were mildly reduced (*p* = 0.049). Additionally, the CARB-PEM group showed higher predicted stress tolerance compared to the NC group (FDR-adjusted *p* = 0.000622) (Figure 4B). It should be noted that BugBase phenotype predictions are computational inferences based on taxonomic composition mapped to reference databases (IMG, KEGG, PATRIC), rather than direct experimental measurements of bacterial phenotypic properties. These predictions reflect potential shifts in community-level phenotypic profiles but cannot be interpreted as confirmed functional or phenotypic changes. Experimental validation through culture-based assays, flow cytometry with Gram staining, or metagenomic/metatranscriptomic approaches would be necessary to confirm these observations.

#### 3.3.2 Metabolic pathway prediction

In addition to phenotypic predictions, this research predicted differences in metabolic pathway abundance in the gut microbiota of Lewis mice after treatment in each group based on the KEGG Pathway database, which is a metabolic pathway database, using PICRUSt2 software.

Compared with the NC group, the PEM-CARB group showed significant differences in the abundance of 24 metabolic pathways, including amino acid metabolic pathway, biosynthesis of other secondary metabolism, and carbohydrate metabolism. And, it is worth noting that the metabolic pathways with differences were all reduced in abundance. However, compared with PEM-CARB group, except for the decreased abundance of glycan biosynthesis and metabolism pathway (*p* = 0.038), there were no statistically significant differences in other metabolic pathways in PEM-CARB-PD-1 group.

Notably, functional predictions were generated using PICRUSt2 and BugBase based on 16S rRNA amplicon data. These results are predictive and hypothesis-generating in nature, and should not be interpreted as direct evidence of functional changes.”

## 4 Discussion

This study explores the effect of the PD-1 inhibitor combined with platinum-containing dual-agent chemotherapy regimen on gut microbiota in Lewis lung cancer model mice and compares it with conventional chemotherapy alone. Our results demonstrated that: (1) chemotherapy significantly altered gut microbiota alpha and beta diversity compared to the tumor-bearing group; (2) the combination of PD-1 inhibitor with chemotherapy was associated with distinct microbiota structural changes compared to chemotherapy alone; and (3) predicted functional profiles suggested differential metabolic pathway representations between treatment groups, although these predictions require experimental validation.

Previous studies have established that patients with higher gut microbiota diversity exhibit better clinical responses to PD-1 inhibitors and longer progression-free survival (Jin et al., 2019; Zhang et al., 2021). However, not only does gut flora diversity have an impact on tumor therapy, but the treatment of tumors likewise affects gut flora, and this alteration in turn affects tumor therapy again.

In this study, both the Shannon and Simpson indices were significantly reduced in the PEM-CARB group relative to the NC group (*p* = 0.015, *p* = 0.007, respectively), consistent with prior reports that chemotherapy induces gut microbiota dysbiosis with reduced diversity (Sougiannis et al., 2019; Xu and Zhang, 2015; Panebianco et al., 2018; Yang et al., 2013). Notably, no further reduction in alpha diversity was observed when PD-1 inhibitor was added to chemotherapy (Shannon: *p* = 0.645; Simpson: *p* = 0.879), suggesting that combination therapy does not exacerbate chemotherapy-induced diversity loss.

In terms of Beta diversity, this study compared the structural similarity of the composition of the groups of colonies using weighted and unweighted Unifrac PCoA. The weighting of Unifrac PCoA was mainly used to differentiate between the effects of high and low abundance of flora in the gut. The lack of statistically significant difference in beta diversity between the CARB-PEM-aPD-1 group and the NC group (weighted UniFrac PCoA: *p* = 0.690; unweighted UniFrac PCoA: *p* = 0.135), while the CARB-PEM group showed significant differences from the Model group (both *p* < 0.001), suggests that the addition of PD-1 inhibitor to chemotherapy did not result in a further detectable alteration in overall microbial community structure comparable to that induced by chemotherapy alone. However, this observation should be interpreted with caution, and equivalence testing or larger sample sizes would be needed to confirm structural similarity.

The relationship between specific microbial taxa and immunotherapy outcomes is highly dependent on tumor type, host genetic background, treatment regimen, geographic factors, and strain-level variation within genera (Derosa et al., 2022). For example: *Akkermansia muciniphila* was associated with favorable PD-1 response in epithelial tumors (Routy et al., 2018)^,^ but its role may differ across cancer types, and excessive mucosal degradation could compromise barrier integrity in certain contexts. Ruminococcus taxa have been reported as both favorable and unfavorable depending on the specific taxa, study population, and cancer type (Gopalakrishnan et al., 2018). Prevotellaceae family members show immunomodulatory effects that vary substantially by taxa and environmental context. Therefore, we refrain from classifying specific genera as “beneficial” or “harmful” and instead describe them as “taxa previously associated with favorable or unfavorable immunotherapy responses in published studies,” acknowledging that our genus-level 16S data cannot resolve the strain-level variation that may underlie these context-dependent effects.

Our data showed that chemotherapy significantly reduced the abundance of the response-favorable taxon Prevotellaceae (*p* = 0.002) and the response-unfavorable taxon Ruminococcus (*p* = 0.028). Compared to chemotherapy alone, the combination therapy further reduced Prevotellaceae abundance (*p* = 0.010) while increasing Ruminococcus abundance (*p* = 0.006). Based on the conceptual framework proposed by Matson et al. (Matson et al., 2018), the net effect of combination therapy on PD-1 efficacy may depend on the balance between response-favorable and response-unfavorable taxa, which warrants quantitative evaluation in future clinical studies.

The phenotypic prediction results suggested an increased phenotypic abundance of Gram-positive bacteria and a decreased phenotypic abundance of Gram-negative bacteria in the combination therapy group relative to the chemotherapy group. Previous studies have reported that an increase in pro-inflammatory Gram-negative bacteria in the gut is associated with systemic and chronic inflammation (Jin et al., 2019). While the predicted shift toward reduced Gram-negative phenotypic abundance in the combination group is noteworthy, it must be emphasized that BugBase provides computational phenotype predictions based on taxonomic composition and reference databases, rather than direct phenotypic measurements. Therefore, the inference that combination therapy does not augment chemotherapy-induced inflammatory effects such as enterocolitis cannot be established from predicted data alone and would require direct experimental validation through intestinal histopathology, inflammatory marker quantification, and immune cell profiling.

Similarly, PICRUSt2-predicted metabolic pathway analysis indicated that the abundance of all differentially represented predicted pathways was reduced in the chemotherapy group compared with the NC group, while nearly no predicted pathways showed statistically significant differences between the combination therapy group and the chemotherapy group. However, PICRUSt2 infers functional gene content from 16S rRNA marker gene data and reference genome databases, which represents predicted metagenomic potential rather than actual metabolic activity. The accuracy of these predictions is inherently limited by database completeness, horizontal gene transfer, and gene expression regulation that cannot be captured by amplicon-based approaches. Consequently, we cannot conclude that the combination therapy does or does not exacerbate chemotherapy-induced intestinal toxicity or metabolic dysfunction based on these predicted data. Direct functional measurements through shotgun metagenomic sequencing, metatranscriptomics, or untargeted metabolomics are required to test this hypothesis.

In summary, the compositional analysis of this study suggests that PD-1 inhibitor combined with platinum-containing dual-agent chemotherapy does not appear to further exacerbate chemotherapy-induced reductions in gut microbiota diversity based on the taxonomic data presented. The combination therapy affects both response-favorable and response-unfavorable taxa, and the final impact on the efficacy of PD-1 inhibitor may depend on the ratio of these two types of flora. Functional predictions from PICRUSt2 and BugBase provide preliminary, hypothesis-generating observations that require rigorous validation through direct functional and experimental approaches.

The interplay between immune checkpoint signaling and gut microbiota extends beyond simple compositional changes. Recent evidence has highlighted the involvement of immune checkpoints in broader inflammatory regulation. Song et al. (Song et al., 2026) reviewed how immune checkpoint molecules, including PD-1/PD-L1, participate in modulating inflammatory responses beyond their canonical roles in tumor immune evasion, with implications for understanding how anti-PD-1 therapy may reshape the gut immune-microbial interface. The dysregulation of immune checkpoint signaling during anti-PD-1 treatment may alter intestinal immune tolerance, potentially contributing to the microbiota structural shifts observed in our study.

From a metabolic perspective, the gut microbiota profoundly regulates host metabolism through diverse pathways. Zhan et al. (Zhan et al., 2026). demonstrated that gut microbiota modulates host metabolic homeostasis through the production of bioactive metabolites, bile acid transformation, and amino acid metabolism, suggesting that treatment-induced microbiota alterations may have metabolic consequences extending beyond the gut lumen Our PICRUSt2 predictions showing reduced metabolic pathway abundance in the chemotherapy group are consistent with this framework, although direct metabolomic validation is needed to confirm actual metabolic perturbation.

Furthermore, short-chain fatty acids (SCFAs), major products of microbial fermentation, serve as critical mediators of microbiota-host inflammatory interactions. Li et al. (Li et al., 2026). comprehensively reviewed how SCFAs modulate intestinal barrier integrity, regulate immune cell differentiation, and suppress excessive inflammatory responses through histone deacetylase inhibition and G-protein-coupled receptor activation The reduction of SCFA-producing taxa such as Lachnospiraceae observed in both our treatment groups may potentially compromise SCFA-mediated anti-inflammatory signaling, although this hypothesis requires confirmation through targeted SCFA quantification and barrier function assessment. These mechanistic considerations collectively highlight that the microbiota structural changes documented in our study may have functional consequences at the intersection of immune regulation, metabolism, and inflammation, warranting integrated multi-omics investigation in future studies.

This study has several important limitations that must be acknowledged. First of all, The absence of additional control groups in the reported analysis. This design cannot fully distinguish the independent effects of tumor burden on microbiota, the independent effects of PD-1 inhibitor monotherapy on microbiota, or potential interactions between chemotherapy and immunotherapy. Our findings are therefore limited to describing microbiota differences associated with combination therapy versus chemotherapy in tumor-bearing mice. Secondly, lack of integrated biological validation. The current study did not include tumor growth curves, terminal tumor weight, body weight monitoring, food intake measurements, colon length, intestinal histopathology (H&E staining), inflammation scoring, or immune cell infiltration analysis (CD8 + T cells, Tregs, MDSCs) within the same cohort. Therefore, we cannot establish whether the observed microbiota changes are causally related to, or merely coincidental with, treatment efficacy or intestinal toxicity. While our parallel studies using the same Lewis lung cancer model have separately assessed intestinal mucosal immunity (IgA + cells, SIgA), inflammatory markers (IFN-*γ*, CASPASE-1, NLRP3, IL-18), and gut barrier function (OCCLUDIN, CLAUDIN), these endpoints were not measured in the specific animals reported here. This represents a significant limitation in our ability to correlate microbiota structural changes with biological outcomes. In addition, although fecal samples were collected at multiple time points during treatment, the present analysis focuses primarily on the end-of-treatment time point. Dynamic longitudinal changes during and after treatment may reveal transient effects or recovery patterns not captured by our analysis. Last but not least, Only male C57BL/6 J mice were used in this study. Sex-specific differences in gut microbiota composition, immune function, and treatment response have been documented. Estrogen and other sex hormones influence both gut microbial community structure and anti-tumor immune responses. Our findings may not be generalizable to female subjects, and future studies should include both sexes.

## Data Availability

The datasets generated and analyzed during the current study are available in the GSA repository under Project number GDDM22050297 https://ngdc.cncb.ac.cn/gsa/s/8z42ieyP, accession number: CRA045001.
