# Supplementary material for: Effect of programmed cell death protein-1 inhibitor combined with platinum-containing dual-agent chemotherapy regimen on gut microbiota in Lewis lung cancer model mice
Source: Front Microbiol. 2026 Jul 10;17:1885048. doi: 10.3389/fmicb.2026.1885048 (PMC13395748; doi:10.3389/fmicb.2026.1885048)
Supplement: Supplementary file 1 [file Supplementary_file_1.docx]

**Supplementary Figure S1**


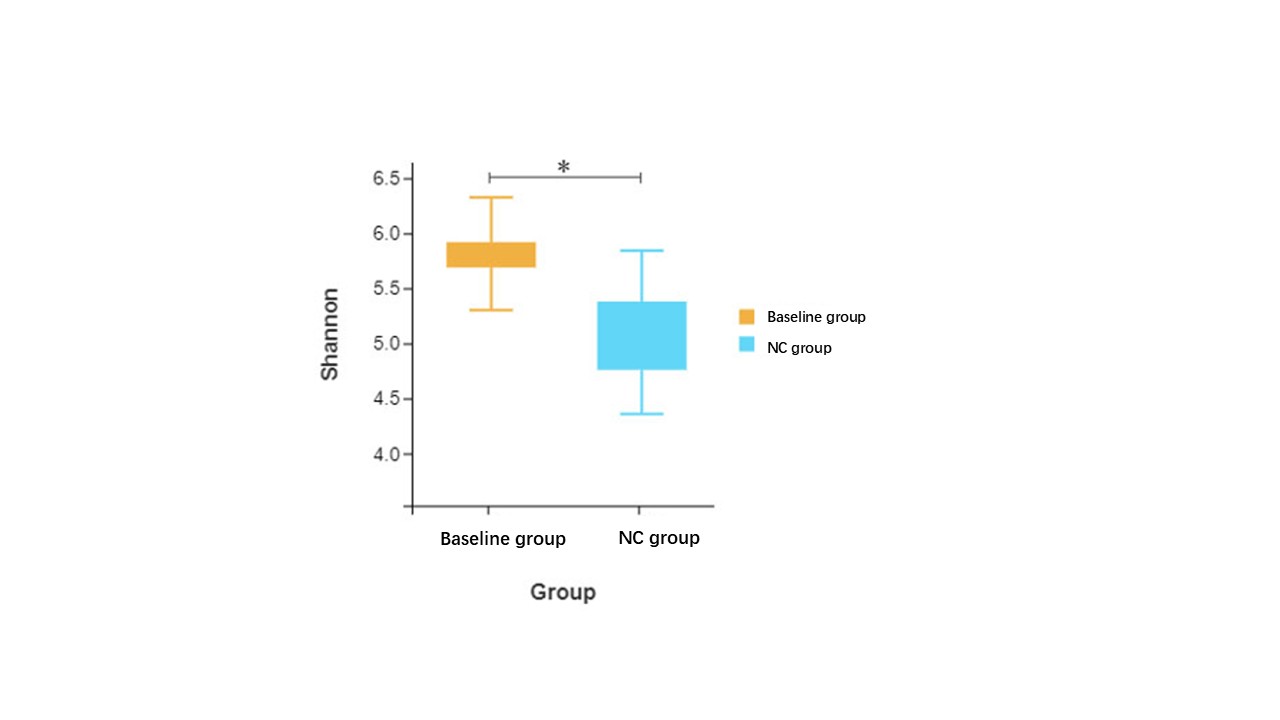


Fig S1.Diversity of the gut microbiota in Lewis mice between baseline group and NC group. Shannon index box chart. The model group had a significantly lower Shannon index than the blank control group. Wilcoxon rank-sum test * P < 0.05, ** P < 0.01, ns P > 0.05.

**Supplementary Figure S2**


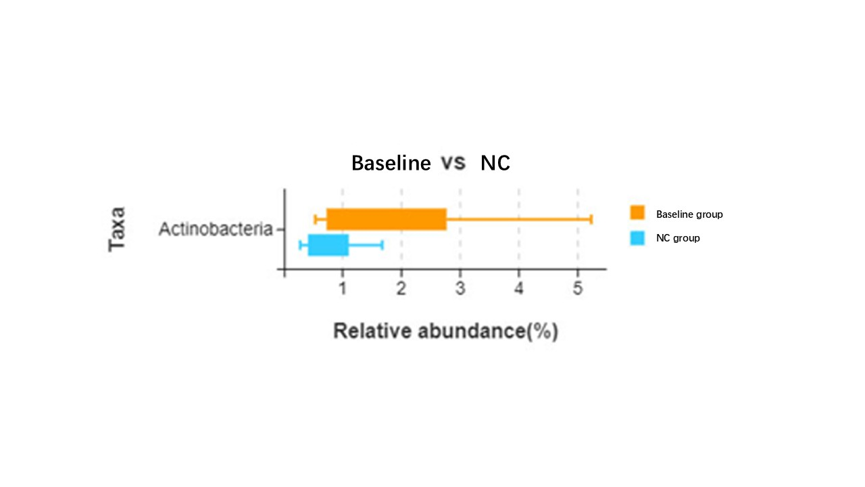


Fig S2.Species distribution stacking maps at the genus level between baseline group and NC group. The abundance of actinomycetes in the model group was significantly lower than in the control group (p = 0.049).

**Supplementary Table S1. Summary of 16S rRNA gene sequencing depth and quality control metrics across experimental groups.**

| **Metric** | **Baseline(n=8)** | **NC (n=8)** | **CARB-PEM (n=8)** | **CARB-PEM-aPD-1 (n=8)** | **P value^a^** |
| --- | --- | --- | --- | --- | --- |
| Raw Reads | 121,827 ± 24,012 | 126,568 ± 5,159 | 126,550 ± 5,560 | 120,327 ± 7,556 | 0.21 |
| Clean Reads | 121,573 ± 23,935 | 126,329 ± 5,112 | 126,307 ± 5,504 | 120,115 ± 7,501 | 0.22 |
| Effective Tags | 101,384 ± 20,431 | 104,507 ± 3,649 | 104,315 ± 4,624 | 99,271 ± 7,520 | 0.18 |
| Effective Rate (%) | 83.16 ± 1.31 | 82.59 ± 0.83 | 82.43 ± 0.72 | 82.44 ± 1.56 | 0.65 |

^a^ Kruskal-Wallis test. All P > 0.05, indicating no significant between-group differences in sequencing depth. Data are presented as mean ± SD.

**Table Legend**

**Supplementary Table S1.** Summary of 16S rRNA gene (V3–V4 region) sequencing depth and quality control metrics. Paired-end sequencing (2 × 250 bp) was performed on the Illumina NovaSeq 6000 platform. Raw Reads: total paired-end reads generated per sample. Clean Reads: reads retained after quality filtering (FASTP, v0.18.0). Effective Tags: final high-quality non-chimeric sequences used for downstream analysis. Effective Rate = (Effective Tags / Raw Reads) × 100%. A total of 4,143,012 effective tags were obtained across 32 samples (overall mean: 103,575 per sample). No significant differences in sequencing depth were observed among groups (Kruskal-Wallis test, all P > 0.05). Per-sample sequencing data are available through the NCBI Sequence Read Archive (accession number: PRJNA×××××). Data are presented as mean ± SD; n = 8 per group.
